# Accelerated forgetting in healthy older samples: Implications for methodology, future ageing studies, and early identification of risk of dementia

**DOI:** 10.1177/17470218221113412

**Published:** 2022-08-01

**Authors:** Terence McGibbon, Ashok Jansari, Jessica Demirjian, Ana Nemes, Adrian Opre

**Affiliations:** 1 Department of Psychology, Goldsmiths, University of London, London, UK; 2 Psychology Department, Babes-Bolyai University, Cluj-Napoca, Romania

**Keywords:** Accelerated long-term forgetting, accelerated forgetting, ageing, forgetting rates, consolidation

## Abstract

Accelerated long-term forgetting (ALF) has been reported in healthy older individuals, and is a possible early marker for risk of developing Alzheimer’s disease (AD). The Verbal Associative Learning and Memory Test (VALMT) addresses methodological weaknesses in existing clinical tests and has detected ALF in epilepsy within an hour. We used VALMT to investigate learning and forgetting in healthy Older participants. Older (60–69 years) and Younger (19–31 years) participants were compared. Using VALMT, unrelated word pairs were learnt to criterion, then cued-recall tested at delays of 5, 30, and 55 min. Unique pairs were tested at each delay. Subjective memory complaints data was gathered, and the Wechsler Memory Scale Logical Memory test (WMS-LM; a standard clinical measure) was administered. VALMT identified a significant difference in delayed recall between Younger and Older groups by 55 min (*d* = 1.32). While “fast-learning” Older participants scored similarly to Younger participants, “slow-learning” Older participants were impaired at all delays. Forgetting rates suggested degradation of memory starts during early synaptic consolidation rather than later system-level consolidation. Increased subjective memory complaints were associated with reduced VALMT scores. By contrast, WMS-LM failed to identify significant differences between any groups, and did not correlate with memory complaints. We conclude that VALMT may be better able than WMS-LM to identify subtle impairments in healthy older adults within a single clinical visit, and VALMT results better reflect subjective experience. Older slow-learners forget faster and report more subjective memory complaints, which may indicate a group at risk of developing AD.

## Introduction

The study of forgetting has a long history in psychology, going back at least to the studies of Ebbinghaus (1885). However, this field has seen increased interest in the past 20 years due to the identification of accelerated long-term forgetting (ALF) as a distinct memory complaint (e.g., Kapur et al., 1997). ALF is typically defined as a disorder in which new information can be learnt and retained normally over at least 30 min (the delay used in standard anterograde memory tests), but is then forgotten at an accelerated rate at longer delays. Initial ALF research focused on people who report being able to remember events and information for several days, but then display an accelerated forgetting which becomes noticeable after a few days to weeks (e.g., Butler et al., 2007). This is of clinical interest as it highlights a class of patients who may perform normally on standard neuropsychological tests of anterograde memory, while still having a genuine memory disorder. It is also of theoretical interest; for example, Butler et al. argue that it provides evidence for secondary consolidation processes, occurring well after initial encoding (Alvarez & Squire, 1994; Nadel & Moscovitch, 1997), which when disrupted can lead to symptoms of late-onset accelerated forgetting.

While early papers in the field had documented ALF in patients with epilepsy with a temporal lobe focus, particularly temporal lobe epilepsy (TLE) or transient epileptic amnesia (TEA), more recent papers have shown that it can also be found in other populations. For example, Gascoigne et al. (2012) have shown that ALF can occur with epilepsy not restricted to the temporal lobes; they have documented ALF in 20 children with idiopathic generalised epilepsy (IGE). More recently, Lah et al. (2017) found ALF in children who had sustained traumatic brain injury (TBI) where, importantly, one of the exclusion criteria was any history of seizures preceding or post the TBI.

ALF has also been identified in healthy older adults. Baddeley et al. (2014) found evidence of ALF in healthy older participants at 6 weeks, using a paradigm based on cued-recall of constrained prose. Manes et al. (2008) found evidence of ALF in older participants who complained of memory problems but who performed normally on standard tests. This group was indistinguishable from matched controls at immediate and 30-min delays, but was significantly impaired at 6 weeks. Manes et al. suggest that we need more sensitive tests to capture these individuals who complain of memory problems in case they are at risk of developing mild cognitive impairment (MCI) and perhaps progressing to Alzheimer’s disease (AD).

Current empirical evidence linking ALF and AD is mixed. In a review, Stamate et al. (2020) found that 7 out of 14 published studies had detected evidence for a link. Concrete evidence was found by Weston et al. (2018) in a study of presymptomatic autosomal dominant AD. This is a familial form of AD, in which carriers of the relevant genetic mutation first display symptoms at relatively predictable ages based on family history. This provides an opportunity to study presymptomatic cognitive change when the remaining time to onset of symptoms can be estimated. Weston et al. found that ALF was detectable in individuals who were on average 7 years away from predicted onset of symptomatic disease. They also found a positive correlation between ALF and subjective memory complaints. They interpret their results as showing that ALF is an early presymptomatic feature of autosomal dominant AD, pre-dating other amnestic deficits, and which might underpin subjective memory complaints. If these results generalise to other forms of AD, then testing for ALF may be valuable diagnostically and in presymptomatic trials. Further support for this argument comes from the finding that a key genetic risk factor for the more common sporadic late-onset AD, apolipoprotein (APOE) ε4, is associated with impaired verbal recall and recognition when this is measured at 7 days, but not at 30 min (Zimmermann & Butler, 2018). The link between subjective memory complaints and AD also supports the argument that subjective complaints can be the first stage of a progressive decline that moves from subjective memory complaints through MCI to AD.

This possibility of using sensitive cognitive tests for early identification of those at risk of MCI/AD is important given the significant prevalence and impact of the disease and the benefits of early diagnosis. Sperling et al. (2011) highlight the need for more sensitive cognitive tests to help track behavioural changes during clinical trials, and as it seems apparent that a combination of biomarker and cognitive tests may prove most effective in early identification of those at risk.

The cause of ALF remains unclear. Disrupted sleep was a common complaint in many of the early documented cases. As sleep has been found to improve performance on both non-cognitive (Peigneux et al., 2004; Walker et al., 2002) and cognitive (Ellenbogen et al., 2006; Fenn et al., 2003) tasks, there was strong reason to believe that this could be a candidate cause for inefficient memory. Mulhert et al. (2010) discussed the possibility that subclinical epileptiform activity during sleep may be a cause of ALF, as prior to medication TEA patients often report amnestic seizures upon awakening. Using a word-pair learning task Mary et al. (2013) found a correlation between ALF and sleep issues in otherwise healthy older people.

More recently, however, some studies have demonstrated that even if abnormal activity during sleep *contributes* to certain aspects of memory dysfunction, this may not be the *central* cause of ALF. Atherton et al. (2014) tested TEA patients’ memory for unrelated word pairs after 12 hr of wakefulness or 12 hr that included a night’s sleep. Contrary to the sleep hypothesis, they found that TEA patients benefitted from sleep just as much as matched controls. In fact, even more surprisingly, the patients performed worse during the wakefulness condition. Similarly, Hoefeijzers et al. (2015) studied recall of word lists in a group of TEA patients across the period of a day and found that there was significant forgetting by 3 hr in the patients.

The inconsistency between different studies is probably driven, at least partly, by methodological differences and weaknesses. In a critical review of existent studies, Elliott et al. (2014) highlighted that no standardised clinical anterograde memory tests go beyond 40 min. They also highlight conditions for learning as a potential issue, because there are a variety of different methods used when teaching material to a particular criterion level. Learning to criterion is a commonly used method to equate initial learning across participants, facilitating comparisons of forgetting curves and helping to avoid ceiling and floor effects. This is typically achieved through increasing the number of learning trials until a particular set criterion is reached. Importantly, there is evidence that equating learning in this manner does not distort the subsequent forgetting curve (Rivera-Lares et al., 2022, building on previous work by Slamecka & McElree, 1983). Irrespective of what the criterion level is set at, if this is applied to the *entire* stimulus set, then the standard procedure is to present the entire set of stimuli repeatedly until the criterion is reached. However, using this procedure, “quickly learnt” individual items will be successfully recalled more times than others during the learning procedure. It has been shown by Roediger and colleagues (Karpicke & Roediger, 2008; Roediger & Karpicke, 2006; Roediger & Smith, 2012) that multiple successful recalls can confer “retrieval practice” and strengthen memories, an effect that would differentially benefit the items that are learnt more quickly, accentuating any initial differences in the learning of individual items, and potentially distorting recall scores.

An additional source of retrieval practice is testing the same material at multiple test delays, for example, words being successfully recalled at one particular time point and then tested again at a later delay. Karpicke and Roediger (2008) have shown that such repeated testing of material significantly improves performance. This means that studies which use the same material at each time point may be masking potential differences between groups at different time points. Baddeley et al. (2021) found that even partial recall of an event can lead to priming of the non-tested elements of the event, resulting in reduced forgetting for all elements, while Stamate et al. (2020) found that patients with AD benefit from repeated retrieval just as much as controls. Using a novel story recall paradigm, Jansari et al. (2010) showed that in a patient with TLE, if the patient was repeatedly tested on the same material, his performance was indistinguishable from that of matched controls by up to 4 weeks after initial learning. However, if he was tested with a different set of stories at each unique time point, he was significantly impaired within 1 day and was functionally amnesic to all material within 2 weeks. This study highlighted the impact of repeated testing of the same material potentially masking any underlying forgetting.

The issue of when the accelerated forgetting starts has also been debated. In a study aimed at addressing methodological issues highlighted both by Elliott et al. (2014) and themselves, Cassel et al. (2016) tested TLE patients using unique material for each time point to avoid retrieval practice effects. They found that TLE patients forgot both verbal and visual information more rapidly than matched healthy controls, and that the forgetting started early. Contrary to suggestions that ALF is driven by a deficit in late consolidation (e.g., Hoefeijzers et al., 2013), they interpreted their findings as evidence for forgetting starting during the early consolidation stage. In later reviews of TLE studies, Cassel and Kopelman (2019) and Mayes et al. (2019) came to differing conclusions. Cassel and Kopelman identified a pattern of early-onset, progressively greater forgetting, and suggest that differences in forgetting patterns reflect a continuum of severity and/or sensitivity. However, Mayes et al. interpret the existing evidence as supporting the existence of ALF as a qualitatively separate memory condition. To help clarify this, further studies which avoid relevant methodological weaknesses are required.

In work with their TLE patient RY, McGibbon and Jansari (2013) attempted to address a number of the methodological issues highlighted above. In their paradigm, the Verbal Associative Learning and Memory Test (VALMT), they taught unrelated word pairs (e.g., TROOP-SHAWL) to their patient RY and matched controls to a criterion of 100% correct and then used cued-recall (e.g., TROOP-?) to evaluate memory. This paradigm addressed previous methodological weaknesses in the following ways: (1) to address the problem of retrieval practice, learning to criterion was applied at the individual word pair level, with presentation of each word pair ceasing once it had been recalled three times successfully; (2) to address the issue of repeated testing, matched but different word pairs were tested at each of the different time points; and (3) to evaluate memory beyond the 40 min highlighted by Elliott et al. (2014), testing was carried out at four discrete intervals, 5, 30, and 55 min and 4 hr after completion of learning. McGibbon and Jansari (2013) found that while their patient was within normal limits at the first two intervals, he was significantly impaired by 55 min.

Previous results for short-story recall (Jansari et al., 2010) showed that RY performed normally at 30 min but was impaired at 24 hr. The fact that impairment on story recall tasks was not found until 24 hr can be explained in at least two ways. First, memory had not been tested at an appropriate delay; story recall had only been tested at 30 min followed by 24 hr, whereas word pair cued recall was tested at 30 and 55 min. Second, the paradigm used could have driven the difference, because the structure of a story may provide enough “scaffolding” to protect a vulnerable memory trace, thereby effectively masking forgetting that is already underway. Therefore, although it *is* important to develop more ecologically valid tests of long-term memory (Baddeley et al., 2014), perhaps for trying to detect accelerated forgetting in the timeframe available to most clinicians, it will be necessary to develop more challenging tasks that address the various methodological issues outlined above. If this is the case the VALMT word pair cued-recall paradigm developed by McGibbon and Jansari (2013) may provide a more sensitive test than that used in many previous ALF studies, and be useful for identifying whether ALF truly reflects late-onset forgetting.

There is also reason to think that the paired associative learning used in the VALMT may make it well suited to identifying memory deficits caused by the early stages of AD. Associative learning is vulnerable to the impact of early stage AD (Sapkota et al., 2017) and it relies heavily on hippocampal and entorhinal cortex regions, which are known to be vulnerable to change in early AD (Coupé et al., 2019; de Rover et al., 2011).

The current study aimed to investigate whether the accelerated forgetting in healthy older adults documented by researchers such as Mary et al. (2013) and Baddeley et al. (2014), which is usually only assessed at long delays, can be detected at shorter delays using the McGibbon and Jansari (2013) VALMT methodology, and how this would be correlated with subjective memory complaints and sleep quality. Initial exploratory pilot and follow-up experiments were conducted. The VALMT was used to compare the performance of a group of healthy older individuals against that of a group of younger participants, comparing retention at 5, 30, and 55 min after first acquiring new information. Results were compared with a standard neuropsychological memory test (WMS-III Logical Memory, Wechsler, 1997), and the relationship to self-reported subjective memory complaints and sleep patterns were investigated.

In the long term, the aim would be to see whether characteristics of the forgetting shown by the Older groups following this paradigm could help to discriminate between healthy ageing and more clinical forms of forgetting, and identify those at risk of developing MCI and AD.

## Pilot study

A pilot study was performed, comparing VALMT performance for a group of 43 Younger participants aged 20–30 years and a group of 26 Older participants aged 65–80 years. Participants were recruited and tested in Romania, and for this study the VALMT was translated into Romanian. Memory was tested at delays of 5, 30, and 55 min, using a unique set of 12 word pairs for each delay. As we were not able to collect any standardised neuropsychological measures of memory or IQ, the detailed results of this pilot are not presented. However, the pilot indicated that the test could reveal statistically significant differences in forgetting rates between education-matched younger and healthy older individuals. The overall analysis showed that while the Younger group showed a very shallow forgetting over the period of 55 min, the Older group showed a much steeper forgetting function. Importantly, when the Older group was separated based on initial learning rate (number of trials required to reach criterion), different patterns of forgetting were revealed. Older individuals who learned rapidly performed similarly to Younger participants, while those who learned more slowly (but to exactly the same criterion) demonstrated lower recall at all delays (5, 30, and 55 min) and a faster rate of forgetting. This relationship with learning performance was therefore examined further in the subsequent main study. Statistics from this pilot are available in Supplementary Appendix A.

## Main study

Our main study was designed to build on the results of the pilot by extending it in four ways. First, the original English version of VALMT, which was designed to investigate ALF in a patient with subclinical temporal lobe epilepsy (McGibbon & Jansari, 2013), was used for the first time to investigate age-related memory decline. Second, building on the previous work indicating a relationship between ALF, subjective memory complaints, and progression to AD (Manes et al., 2008; Weston et al., 2018), self-report measures of memory functioning were taken. Third, to address concurrent validity, results from both the VALMT and self-report measures were compared with a standard neuropsychological measure commonly used to assess memory functioning (WMS-LM test; Wechsler, 1997). Fourth, following evidence from studies such as Mary et al. (2013) that disrupted sleep can impact memory, we explored sleep quality using a self-report measure.

We predicted that our Younger participants would perform better than Older participants at delayed recall, that the slow learners among our Older participants would show increased evidence of ALF and a higher level of memory complaints, that the VALMT would be a more sensitive measure of ALF than the WMS-LM, and that ALF would be positively correlated with disrupted sleep patterns.

## Method

### Participants

Two groups of participants were assessed: 30 Younger participants aged 19–31 years (21 F, 9 M; *M*_age_: 24.83, *SD*: 2.87) were compared with 30 Older participants aged 60–69 years (20 F, 10 M; *M*_age_: 63.97, *SD*: 2.54). All participants reported that they were healthy and free from any psychological or medical condition that could have an impact on their memory.

### Materials and measurements

#### Pre-morbid IQ assessment

All participants completed the Wechsler Test of Adult Reading (WTAR; Holdnack, 2001) to provide a pre-morbid measure of IQ.

#### Neuropsychological memory assessment

To provide a comparison for the VALMT and a standard neuropsychological measure of memory the Wechsler Memory Scale-III UK Edition Logical Memory test was administered (Wechsler, 1997). The Adult Battery form (ages 16–69 years) was used for all groups. To test for immediate and delayed (30 min) recall the Logical Memory I (LMI) and II (LMII) subtests were administered to all participants, respectively. LMI and LMII are composed of the same two stories, each consisting of 25 items. One point was given for each correctly recalled item. The raw scores of the two stories were combined to form a total raw score (max = 50).

#### VALMT Word pair cued-recall task

McGibbon and Jansari’s (2013) VALMT word pair cued-recall paradigm was used. A subset of the words in the original McGibbon and Jansari (2013) study were used, which were matched for familiarity, concreteness, imageability, and frequency. All words were nouns, two syllables and 4–6 letters long. These were used to compose 36 word pairs. Words were randomly assigned to pairs. Words in any pairs with obvious semantic relationships were re-paired.

The stimulus set consisted of three lists of word pairs (corresponding to the three testing delays under investigation, i.e., 5, 30, and 55 min). Each list consisted of 12 word pairs (e.g., *TROOP—SHAWL*). At learning the material from the 3 lists was interleaved (see section “Procedure,” below). The learning procedure was computer-based, using the same custom-written software used in the original study. Memory was tested using simple paper and pencil.

#### Subjective sleep quality

Sleep quality was assessed using the Pittsburgh Sleep Quality Index (PSQI; Buysse et al., 1989). The PSQI is a self-reported questionnaire composed of nine questions which assess the quality of sleep during the past month across seven domains, with the answers being combined to obtain a global PSQI score for each participant (range 0–21), with a score of 5 or greater indicating *poor sleep quality*.

#### Subjective memory complaints

To investigate whether participants subjectively report having memory problems, the Memory Complaint Scale (MCS) was administered to all participants (Vale et al., 2012). The questionnaire consists of seven questions. Following standard MCS procedures the scores of all seven questions were summed to form a total MCS score between 0 and 14 and each participant was placed into one of four ordinal categories based on severity of memory complaints: No memory complaints (MCs: 0–2), Mild MCs (3–6), Moderate MCs (7–10), and Severe MCs (11–14). Finally, to compare our data with those of Manes et al. (2008), additional custom MCS dichotomous categories were defined: non-complainers (0–2) and complainers (3–14).

### Procedure

All testing was performed in a quiet room, either at Goldsmiths, University of London or in the participant’s home, with the experimenter as the only other person present.

Participants first completed the WTAR assessment, the self-administered MCS and PSQI questionnaires, and provided demographic information including age and education.

**Figure 1.**
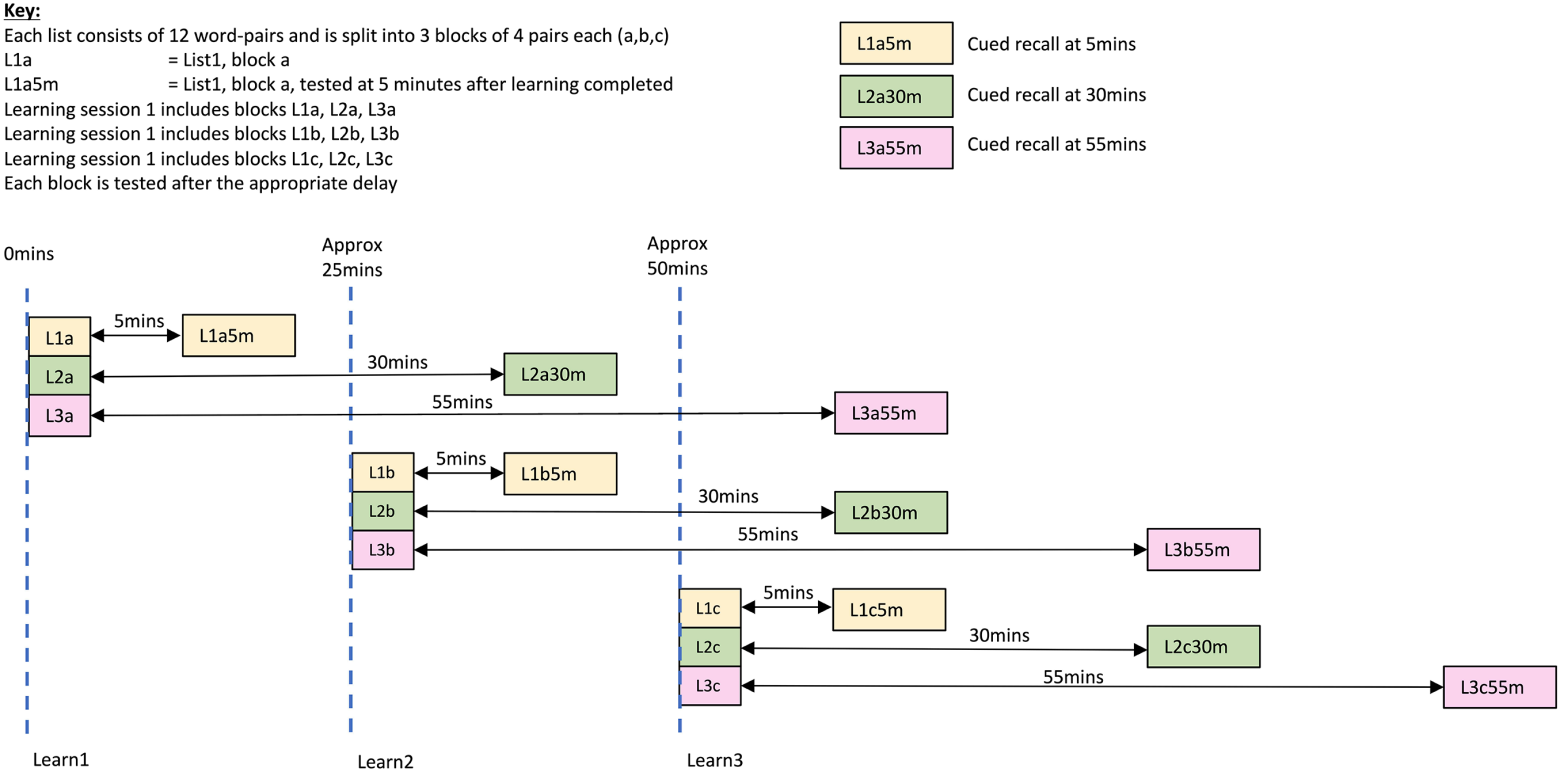
Learning and testing schedule.

Next the VALMT was administered. To avoid fatigue, the 36 word pairs word were split across three learning periods, with 12 word pairs learnt during each period. The pairs from the three learning lists were split equally across the three learning periods, so the stimuli for each consisted of 12 pairs total, 4 from each list (4 pairs each from the 5, 30, and 55 min lists; refer to Figure 1). Within each learning period the material from the three lists was interleaved so that any changes in strategy, or loss in concentration, or tiredness/stress, would impact all three lists equally.

Initially, each pair (e.g., TROOP—SHAWL) was presented once, for 7 s, in a fixed sequence. Once all pairs had been presented, their sequence was randomised. Participants were presented with the first word of a pair (e.g., TROOP-???) and were required to type in the second word of the pair. Immediate feedback was then provided, which included display of the correct pairing for 2 s (“Correct. The correct pairing is: . . .” *or* “Incorrect. The correct pairing is: . . .”). After displaying all pairs, the process was repeated, using a new random order. This process was repeated in a continual loop with the software having been written such that once any individual pair had been answered correctly three times it was removed from the list. Once all pairs had been removed from the list (100% learning criterion) the learning session was complete.

Each learning period was followed by a 5-min rest, and then a test period. During the rest period, participants performed a distraction task (pencil and paper maze completion) to prevent rehearsal. Testing of the relevant material from each learning period was carried out at the three time points (5, 30, and 55 min); to ensure the correct delays, there were multiple tests (see Figure 1). Where material from multiple lists was being tested at the same time, the pairs were interleaved.

Assessment was carried out using pen and paper; the participant was provided with a sheet that contained the first word of each studied pair, and was asked to fill in the appropriate accompanied word. In total the learning and testing periods, including distractor tasks, lasted 1.5–2 hr, depending on each individual participant’s learning speed.

To avoid interference with the VALMT, all participants were seen again on a different day for administration of the WMS LMI and LMII subtests. During the 30-min rest period between LM1 and LM11 tests, participants performed a non-verbal distraction task (pencil and paper maze completion) to prevent rehearsal.

### Ethics

The research was approved by the Research Ethics Board of Goldsmiths, University of London. The procedure was explained to participants and their written consent obtained before conducting any testing. Participants were informed that they could withdraw from the study at any point without giving a reason and were provided with a written leaflet containing a short debriefing of research aim and contact details.

### Statistical analysis

Statistical analyses were carried out using SPSS v24 and JASP v0.16.1. Data were checked for normality, homogeneity of variance, and for sphericity as appropriate. Where data were not normally distributed Mann Whitney U (MWU) tests were used to compare means. Where the assumption of homogeneity of variance was violated, Welch’s F ratio was used and *t*-tests analysed assuming unequal variance. Where sphericity was violated, the Greenhouse–Geisser correction for nonsphericity was applied. Overall, analyses and interaction effects were investigated using mixed analyses of variance (ANOVA). Comparisons of means across groups at specific delays were performed using one-way ANOVAs and independent sample *t*-tests. Comparisons of forgetting rates within groups were performed using paired-sample *t*-tests. All *t*-tests were 2-tailed. Bonferroni adjusted LSD post hoc tests were used to investigate significant ANOVA results. Effect size was estimated using η_p_^2^, Pearson’s *r*, and Cohen’s *d* as appropriate. Where multiple comparisons were conducted using *t*-tests or correlations, the *p*-values for any significant results were Bonferroni adjusted by multiplying by the number of comparisons and retaining the standard significance level (.05). JASP was used to perform equivalent Bayesian tests where available, using default priors throughout, and the resulting Bayes factors (BF) are reported. BF quantify the degree to which data support either the null hypothesis (for a 2-tailed test, no effect or group difference exists) or the alternative hypothesis (for a 2-tailed test, an effect or group difference does exist). We report BF for the alternative hypothesis throughout, denoted BF_10_. Following the descriptive classifications of Lee and Wagenmakers (2014), BF_10_ between 1 and 3 provide *anecdotal evidence* for the alternative hypothesis, values between 3 and 10 provide *moderate evidence*, values between 10 and 30 provide *strong evidence*, values between 30 and 100 provide *very strong evidence*, and values above 100 provide *extreme evidence*. BF_10_ values below 1 provide evidence for the null hypothesis in equivalent bands (*anecdotal*: 1–1/3, *moderate*: 1/3–1/10, *strong*: 1/10–1/30, *very strong*: 1/30–1/100, *extreme*: <1/100).

## Results

### Demographics

Groups were matched on all demographic variables (see Table 1).

**Table 1.** Demographic information as a function of age group.

Factor	Category	Younger *N* (%) or *M* (*SD*)	Older *N* (%) or *M* (*SD*)	Statistical test
Gender	Male	9 (30%)	10 (33%)	χ^2^(1) = 0.08, *p* = .78, BF_10_ = 0.35
	Female	21 (70%)	20 (67%)	
Education	Less than high School	3 (10%)	4 (13%)	χ^2^(3) = 0.74, *p* = .86, BF_10_ = 0.09
	High school	9 (30%)	10 (33%)	
	Bachelor degree	11 (37%)	8 (27%)	
	Graduate degree	7 (23%)	8 (27%)	
WTAR Predicted IQ		100.23 (5.16)	100.17 (5.42)	*t* (58) = 0.05, *p* = .961, BF_10_ = 0.26

WTAR: Wechsler Test of Adult Reading (WTAR; Holdnack, 2001).

### Learning performance

An independent sample *t*-test was used to compare the mean number of trials needed to reach criterion (mean across the 3 learning periods). The Older group took significantly more trials to reach criterion (*M_Older_* = 69.77 trials, *M*_Younger_ = 49.40 trials; *t*(58) = 3.02, *p* = .004, *d* = 0.79, BF_10_ = 10.45).

The variance was greater for the Older group than the Younger group, although this failed to reach significance (Older: *SD* = 35.4; Younger: *SD* = 10.35; Levene’s test for equality of variances not violated, *p* = .10).

To investigate the role of learning performance identified in the pilot study, the Older group was divided using a median split into those who required fewer trials (Fast_Older, *n* = 15) and those who required more trials (Slow_Older, *n* = 15) for some of the later analyses. The split point was set at the median value, 56 trials.

The Fast_Older group took more trials than the Younger group to reach criterion, but the difference was small and not significant (*M*_Fast_Older_ = 51.40 trials, *M*_Younger_ = 49.40 trials; *MWU*(43) = 168.0, *p* = .17 *r* = .20, BF_10_ = 0.40), while the Slow_Older group took significantly more trials than the Younger group (*M*_Slow_Older_ = 88.13 trials, *M*_Younger_ = 49.40 trials; *MWU*(43) = 26.5, Bonferroni adjusted *p* < .001, *r* = .71, BF_10_ = 92.63).

A comparison of the two Older groups showed no significant difference in mean age (*M*_Fast_Older_ = 64.07 years, *M*_Slow_Older_ = 63.87 years; *t*(28) = 0.21, *p* = .83, *d* = 0.08, BF_10_ = 0.35), gender (χ^2^(1) = 0.60, *p* = .43, BF_10_ = 0.59), education (χ^2^(3) = 2.9, *p* = .41, BF_10_ = 0.55), or IQ (*t*(28) = 0.70, *p* = .489, *d* = 0.26, BF_10_ = 0.41).

### VALMT delayed cued-recall performance

**Figure 2.**
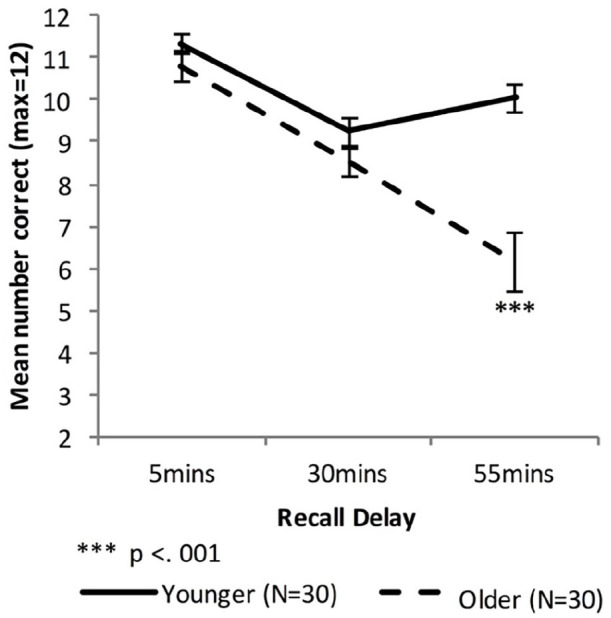
Mean VALMT recall scores as a function of time delay and group (error bars represent one standard error).

**Figure 3.**
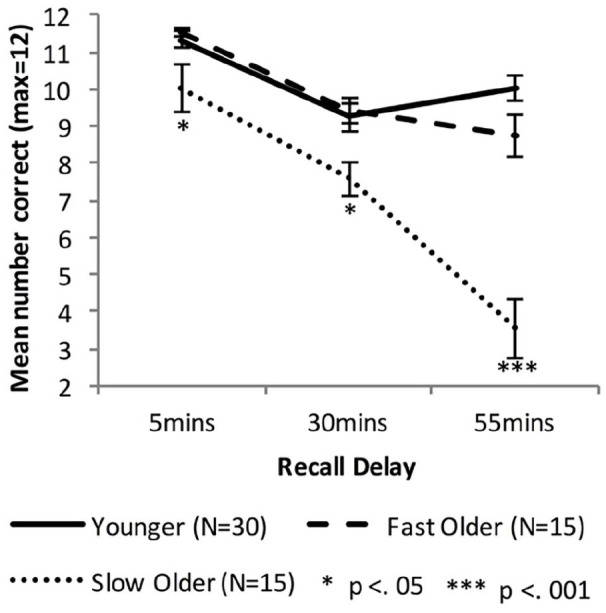
Mean VALMT recall scores as a function time delay and group, separating the Older group into two groups based on initial learning (error bars represent one standard error).

Figure 2 shows the delayed recall performance of the Younger group and the combined Older group, while Figure 3 shows the performance for the Fast_Older, Slow_Older and Younger groups.

#### Combined Older group

A mixed-factors ANOVA with within-subjects factor delay (5 vs. 30 vs. 55 min) and between-subjects factor age (Younger vs. Older) was used to analyse cued recall performance across all delay intervals.

There were significant main effects of Age, *F*(1, 58) = 13.72, *p* < .001, η_p_^2^ = .19, BF_10_ = 3.00 × 10^7^, and Delay, *F*(1.44, 83.48) = 57.55, *p* < .001, η_p_^2^ = .50, BF_10_ = 2.50 × 10^14^, and a significant interaction between Age and Delay, *F*(1.44, 83.48) = 21.47, *p* < .001, η_p_^2^ = .27, BF_10_ = 2.87 × 10^6^, indicating that compared with the Younger group the Older group had an overall lower level of recall (Marginal means: *M*_*Older*_ = 8.47 pairs, *M*_Younger_ = 10.20 pairs) and a higher forgetting rate.

To compare cued recall performance across Younger and Older groups at each time point three independent sample *t*-tests were used. There was no significant difference at 5 or 30 min (5 min: *M_Older_* = 10.77 pairs, *M*_Younger_ = 11.33 pairs, *t* (58) = 1.40, *p* = .17, *d* = 0.37, BF_10_ = 0.59; 30 min: *M_Older_* = 8.50 pairs, *M*_Younger_ = 9.23 pairs, *t* (58) = 1.56, *p* = .13, *d* = 0.41, BF_10_ = 0.72). However, the Older group had significantly lower levels of recall compared with the Younger group at 55 min (55 min: *M_Older_* = 6.13 pairs, *M*_Younger_ = 10.03 pairs, *t* (43.0) = 5.03, Bonferroni adjusted *p* < .001, *d* = 1.32, BF_10_ = 3,137).

Forgetting rates were calculated as amount of information lost between two consecutive time points relative to the amount that had been recalled at the earlier of the two time points. Therefore, the “early” forgetting rate (that between the 5- and 30-min time points) was calculated as *[5* *min score – 30* *min score]/5* *min score*, and the “late” forgetting rate (that between the 30 and 55 min time points) was calculated as *[30* *min score – 55* *min score]/30* *min score*. Independent samples *t*-tests found no significant difference in early-forgetting rate (*M_Older_* = 0.21, *M*_Younger_ = 0.19; *t*(58) = 0.673, *p* = .50, *d* = 0.18, BF_10_ = 0.32), while the Older group had a significantly higher late-forgetting rate (*M_Older_* = 0.30, *M*_Younger_ = −0.11; *t*(47.7) = 4.83, Bonferroni adjusted *p* < .001, *d* = 1.27, BF_10_ = 1,667).

To investigate changes in forgetting rates, paired sample *t*-tests were used to compare early and late forgetting rates for each group separately. There was a significant difference between early and late rates for the Younger group (*M*_early_ = 0.187, *M*_late_ = −0.114; *t*(29) = 4.64, Bonferroni adjusted *p* < .001, *d* = 1.53, BF_10_ = 365) but not for Older group (*M*_early_ = 0.208, *M*_late_ = 0.301; *t*(29) = 1.21, *p* = .238, *d* = 0.32, BF_10_ = 0.37).

#### Fast and slow learning Older groups

Equivalent analyses of cued recall performance across all delay intervals were performed with the Older group split into Fast and Slow learning groups using a median split on number of trials required during learning. A mixed-factors ANOVA with within-subjects factor Delay (5 vs. 30 vs. 55 min) and between-subjects factor Group (Younger vs. Fast_Older vs. Slow_Older) identified significant main effects of Delay, *F*(1.6, 88.9) = 87.41, *p* < .001, η_p_^2^ = .61, BF_10_ = ∞, and Group, *F*(2, 57) = 23.22, *p* < .001, η_p_^2^ = 0.45, BF_10_ = ∞, and a significant interaction, *F*(3.1, 88.9) = 21.52, *p* < .001, η_p_^2^ = .43, BF_10_ = 5.51 × 10^10^. Bonferroni adjusted post hoc tests found no significant difference between the Younger and Fast_Older group (*p* = 1.00, BF_10_ = 0.28), but significant differences between the Younger and Slow_Older (*p* < .001, BF_10_ = 1.06 × 10^7^) and between the Fast_Older and Slow_Older (*p* < .001, BF_10_ = 1,360).

Recall scores at each individual delay were compared using one-way ANOVAs, and significant results investigated using Bonferroni adjusted post hoc tests for pairwise comparisons. There was a significant difference between the means at all three delays (5 min: *F*(2,57) = 5.143, *p* = .009, η_p_^2^ = .15, BF_10_ = 5.08; 30 min: *F*(2,57) = 5.47, *p* = .007, η_p_^2^ = .16, BF_10_ = 6.41; 55 min: *F*(2,57) = 38.42, *p* < .001, η_p_^2^ = .57, BF_10_ = 2.66 × 10^8^). At all delays the Slow_Older group performed statistically below the Younger (5 min: *p* = .018, *d* = 0.79, BF_10_ = 3.50; 30 min, *p* = .012, *d* = 0.88, BF_10_ = 5.99; 55 min: *p* < .001, *d* = 2.69, BF_10_ = 6.67 × 10^7^) and Fast_Older groups (5 min: *p* = .019, *d* = 0.86, BF_10_ = 2.61; 30 min, *p* = .017, *d* = 1.18, BF_10_ = 12.36; 55 min: *p* < .001, *d* = 1.89, BF_10_ = 938). There was no significant difference between the Younger and Fast_Older groups’ performance at any delay (5 min: *p* = 1.00, *d* = 0.20, BF_10_ = 0.37; 30 min, *p* = 1.00, *d* = .09, BF_10_ = 0.32; 55 min: *p* = .264, *d* = .64, BF_10_ = 1.54).

Forgetting rates were compared across groups using one-way ANOVAs, and significant results investigated using Bonferroni adjusted post hoc tests for pairwise comparisons. For early forgetting the difference between the three group means was not significant, *F*(2,57) = 0.83, *p* = .44, η_p_^2^ = .03, BF_10_ = 0.26, whereas the difference was significant for late forgetting, *F*(2,57) = 26.20, *p* < .001, η_p_^2^ = .48, BF_10_ = 1.25 × 10^6^. Post hoc tests comparing the Slow_Older group with the Younger and Fast_Older groups found the Slow_Older had a significantly higher late forgetting rate (Younger: *p* < .001, *d* = 1.44, BF_10_ = 6.04 × 10^5^; Fast_Older: *p* < .001, *d* = 1.47, BF_10_ = 67.55), while there was no significant difference between the Younger and Fast_Older groups’ late forgetting rates (*p* = .174, *d* = 0.21, BF_10_ = 2.18).

To investigate changes in forgetting rates for the Older groups paired-sample *t*-tests were used to compare early and late forgetting rates for each group separately. There was no significant difference between early and late rates for the Fast_Older group (*M*_early_ = 0.184, *M*_late_ = 0.062; *t*(18) = 1.57, *p* = .14, *d* = 0.64, BF_10_ = 0.72). However, the difference was significant for the Slow_Older group (*M*_early_ = 0.23, *M*_late_ = 0.54; *t*(14) = 2.83, Bonferroni adjusted *p* = .026, *d* = 1.11, BF_10_ = 4.41).

#### Relationship between learning period and recall at 55-min delay

The VALMT procedure includes interleaving of learning and testing. This ensures that any changes in strategy, or loss in concentration, or fatigue, or stress will impact the learning of all lists equally, and makes it feasible to complete the entire process within a single visit. However, it has the potential to create interference occurring between learning and test that may negatively impact delayed recall. Any such effect would be greatest for the pairs tested at 55 min. Referring to Figure 1, for these 55-min pairs, the items learnt in the first learning period, Learning Period 1, and then tested after 55 min (L3a pairs) have the greatest potential to experience interference, while those learnt in Learning Period 2 (L3b pairs) should encounter less interference, and finally those learnt in Learning Period 3 (L3c pairs) should encounter the least interference. Using this rationale, if interference was impacting results we would expect to see an inverse pattern in recall scores, with L3a pairs getting the lowest recall score, L3b pairs the second highest, and L3c pairs the highest. The mean recall scores for each of these three sets are summarised in Table 2.

**Table 2.** 55-min delayed recall as a function of group and period in which pairs were learnt, separating the Older group into two groups based on initial learning.

Group	Learning Period 1 (L3a) *M* (*SD*)	Learning Period 2 (L3b) *M* (*SD*)	Learning Period 3 (L3c) *M* (*SD*)
Younger (*N* = 30)	3.00 (1.14)	3.47 (0.90)	3.57 (0.82)
Fast_Older (*N* = 15)	2.60 (1.12)	3.13 (1.25)	3.00 (0.93)
Slow_Older (*N* = 15)	1.20 (1.15)	1.27 (1.38)	1.07 (1.16)

Applying a mixed-factors ANOVA with within-subjects factor Learning Period (Learning Period 1[L3a] vs. Learning Period 2[L3b] vs. Learning Period 3[L3c]) and between-subjects factor Group (Younger vs. Fast_Older vs. Slow_Older) identified a significant main effect of Group, *F*(2,57) = 38.43, *p* < .001, η_p_^2^ = .57, BF_10_ = 2.27 × 10^8^, but no significant effect of Learning Period, *F*(2,114) = 2.39, *p* = .10, η_p_^2^ = .04, BF_10_ = 0.86, and no significant interaction, *F*(4,114) = .86, *p* *=* .49, η_p_^2^ = .03, BF_10_ = 0.28. While no significant effect of learning period was found, the Bayes factor indicates the data provide only anecdotal evidence for a lack of effect (the null hypothesis). However, the Bayes factor for the interaction shows strong evidence for the lack of an interaction, indicating that interference does not impact our groups differently and therefore is unlikely to be the cause of observed group differences in recall.

### WMS logical memory story recall performance

#### Standardised assessment

All participants scores were compared with the WMS-LM normative data, which showed that none of the participants were impaired.

#### Recall performance

**Figure 4.**
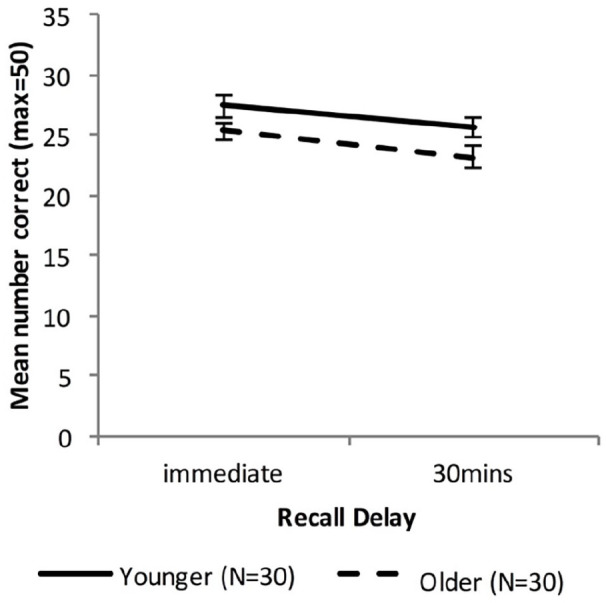
Mean WMS-LM recall scores as a function of time delay and group (error bars represent one standard error).

**Figure 5.**
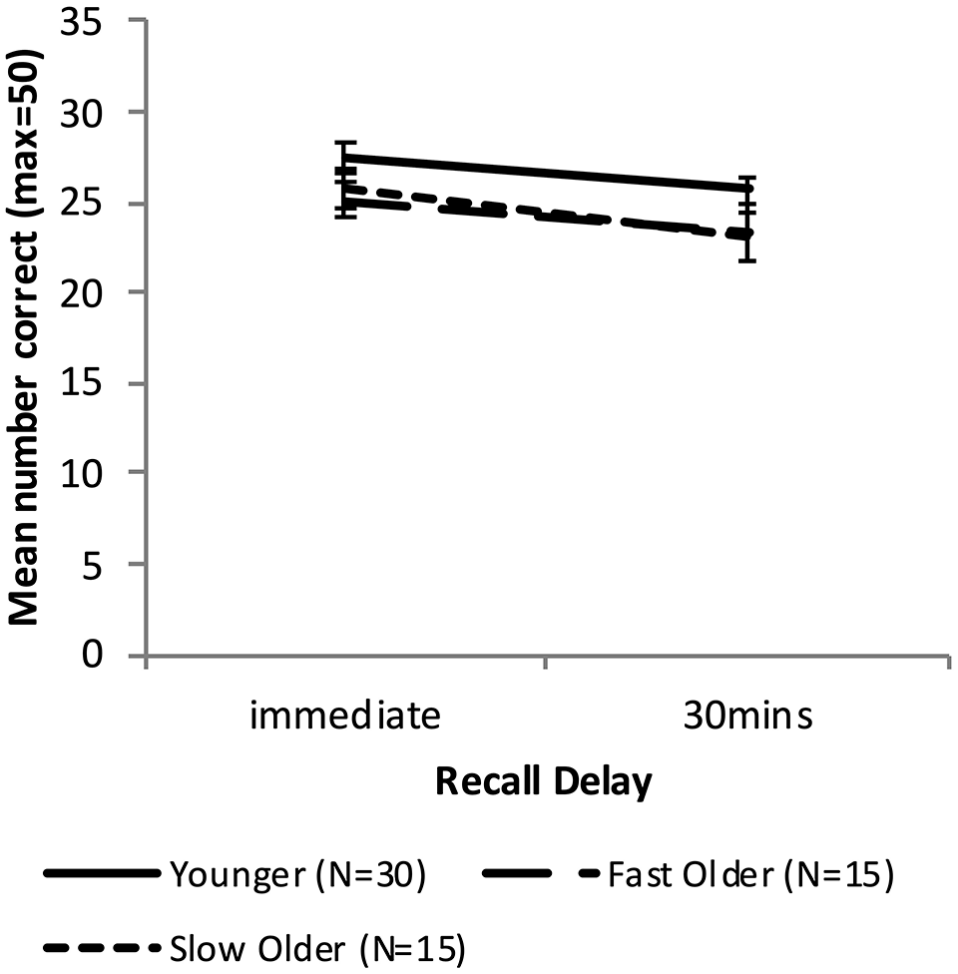
Mean WMS-LM recall scores as a function time delay and group, separating the Older group into two groups based on initial learning (error bars represent one standard error).

Figure 4 shows the free recall performance of the Younger group and the combined Older group, while Figure 5 shows the performance for the Younger, Fast_Older and Slow_Older groups (please note that these groupings are based on VALMT learning performance as above).

#### Combined Older group

A mixed-factors ANOVA with within-subjects factor Delay (immediate vs. 30 min) and between-subjects factor Age (Younger vs. Older) was used to analyse cued recall performance across all delay intervals.

There was a significant main effect of Delay, *F*(1, 58) = 18.49, *p* < .001, η_p_^2^ = .24, BF_10_ = 272. The main effect of Age approached significance, *F*(1, 58) = 4.01, *p* = .05 η_p_^2^ = .065, BF_10_ = 1.20, and there was no significant interaction between Age and Delay, *F*(1, 58) = 0.22, *p* = .64, η_p_^2^ = .004, BF_10_ = 0.63, indicating that compared with the Younger group the Older group had an overall lower level of recall (Marginal means: *M_Older_* = 24.3, *M*_Young_ = 26.5) but did not differ in forgetting rate.

To compare recall performance across Younger and Older groups at each time-point two independent sample *t*-tests were used. There was no significant difference in immediate or 30-min recall (immediate: *M_Older_* = 25.4, *M*_Younger_ = 27.4, *t* (58) = 1.81, *p* = .075, *d* = 0.48, BF_10_ = 1.03; 30 min: *M_Older_* = 23.2, *M*_Younger_ = 25.7, *t* (58) = 1.89, *p* = .063, *d* = 0.50, BF_10_ = 1.17).

Forgetting rates were calculated as [immediate recall – 30-min recall)]/immediate recall. An independent samples *t*-test found no significant difference in forgetting rate (*M_Older_* = .085, *M*_Younger_ = .060; *t*(58) = 0.634, *p* = .53, *d* = 0.17, BF_10_ = 0.31).

#### Fast and slow learning Older groups

Equivalent analyses were performed with the Older group split into the Fast and Slow learning groups identified earlier. A mixed-factors ANOVA with within-subjects factor Delay (immediate vs. 30 min) and between-subjects factor Group (Younger vs. Fast_Older vs. Slow_Older) identified a significant main effect of Delay, *F*(1, 57) = 17.67, *p* < .001, η_p_^2^ = .24, BF_10_ = 257, but no significant main effect of Group, *F*(2, 57) = 1.99, *p* = .15, η_p_^2^ = .065, BF_10_ = 0.59, and no significant interaction, *F*(2, 57) = 0.29, *p* = .75, η_p_^2^ = .01, BF_10_ = 0.28.

Recall scores at each delay were compared using one-way ANOVAs. There was no significant difference between the means at either delay (immediate: *F*(2, 57) = 1.71, *p* = .191, η_p_^2^ = .06, BF_10_ = 0.51; 30 min: *F*(2, 57) = 1.77, *p* = .179, η_p_^2^ = .06, BF_10_ = 0.54).

Forgetting rates were compared across groups using a one-way ANOVA, with no significant difference found, *F*(2, 57) = 0.40, *p* = .673, η_p_^2^ = .01, BF_10_ = 0.19.

### Subjective sleep quality

**Figure 6.**
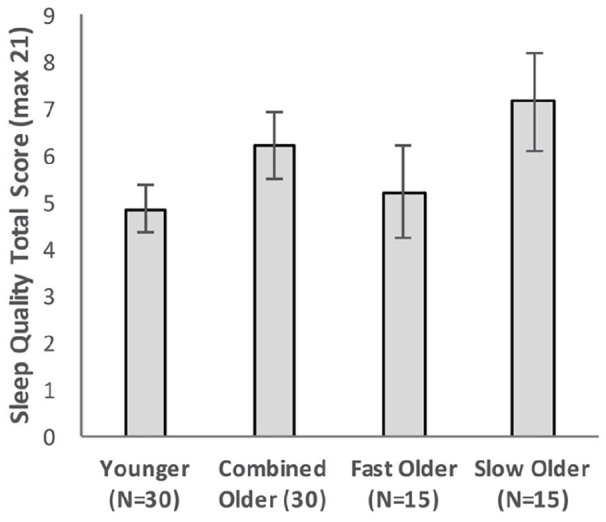
Sleep quality total score as a function of Group, with Older participants shown as a combined group and separated into two groups based on initial learning (error bars represent one standard error).

Figure 6 shows the sleep quality scores of the Younger group, the combined Older group, and the Older group split into Fast_Older and Slow_Older as described above.

#### Combined Older group

Sleep quality scores for Younger and Older groups were compared using an independent sample *t*-test. There was no significant difference (*M_Older_* = 6.17, *M*_Younger_ = 4.83, *t*(52.39) = 1.49, *p* = .14, *d* = 0.39, BF_10_ = 0.67).

#### Fast and slow learning Older groups

Sleep quality scores were compared using a one-way ANOVA. There was no significant difference between the means of the Younger, Fast_Older, and Slow_Older groups, *M*_Younger_ = 4.83, *M*_Fast_Older_ = 5.20, *M*_Slow_Older_ = 7.13, *F*(2, 57) = 2.35, *p* = .104, η_p_^2^ = .08, BF_10_ = 0.77.

### Subjective memory complaints

**Figure 7.**
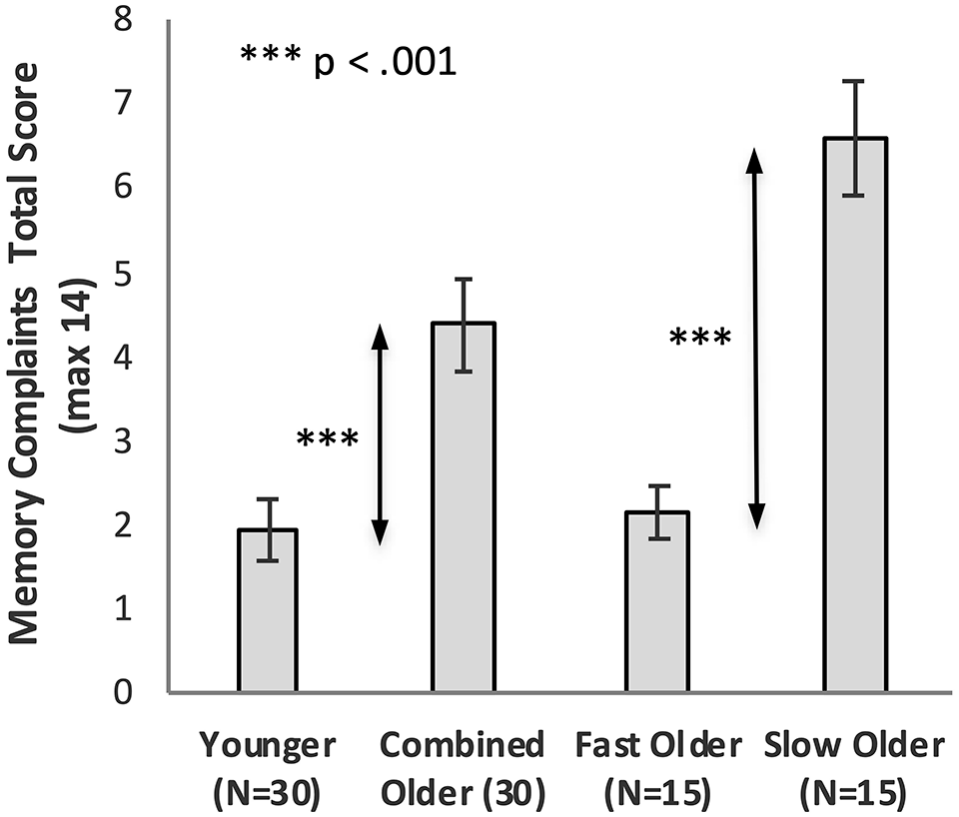
Memory complaints total score as a function of Group, with Older participants shown as a combined group and separated into two groups based on initial learning (error bars represent one standard error).

Figure 7 shows the total MCS scores of the Younger group, the combined Older group, and the Older group split into Fast_Older and Slow_Older groups.

#### Combined Older group

Memory complaint scores for Younger and Older groups were compared using an independent sample *t*-test. The Older group had significantly higher complaints, *M_Older_* = 4.37, *M*_Younger_ = 1.93, *t* (48.6) = 3.73, *p* = .001, *d* = 0.98, BF_10_ = 61.79.

#### Fast and slow learning Older groups

Memory complaint scores were compared using a one-way ANOVA. There was a significant effect of Group on memory complaints, *F*(2, 57) = 30.72, *p* < .001, η_p_^2^ = .52, BF_10_ = 9.21 × 10^6^. Bonferroni adjusted post hoc tests confirmed there was no significant difference between the Younger and Fast_Older groups (Younger vs. Fast_Older *p* = 1.00, *d* = 0.11, BF_10_ = 0.33), while there was a significant difference between the Slow_Older and both the Younger and Fast_Older groups (Younger vs. Slow_Older *p* < .001, *d* = 2.16, BF_10_ = 3.74*10^5^; Fast_Older vs. Slow_Older *p* < .001, *d* = 2.18, BF_10_ = 6,514).

Table 3 summarises the MCS scores for each group when categorised into four ordinal categories (see section “Method”); 80% of Younger and 67% of Fast_Older were non-complainers, whereas 87% of the Slow_Older were complainers (20% Mild and 67% Moderate complainers).

**Table 3.** Distribution of memory complaints across age groups.

Memory complaints category	Younger *N* (%)	Fast_Older *N* (%)	Slow_Older *N* (%)
None	24 (80%)	10 (67%)	2 (13%)
Mild	4 (13%)	5 (33%)	3 (20%)
Moderate	2 (7%)	0 (0%)	10 (67%)
Severe	0 (0%)	0 (0%)	0 (0%)

#### Relationship between subjective memory complaints and VALMT scores

There was a significant correlation between memory complaints and VALMT recall score at 55 min for the Older group (*r* = −.74, Bonferroni adjusted *p* < .001, BF_10_ = 7,427) but not for the Younger group (*r* = −.09, *p* = .62, BF_10_ = 0.26).

**Figure 8.**
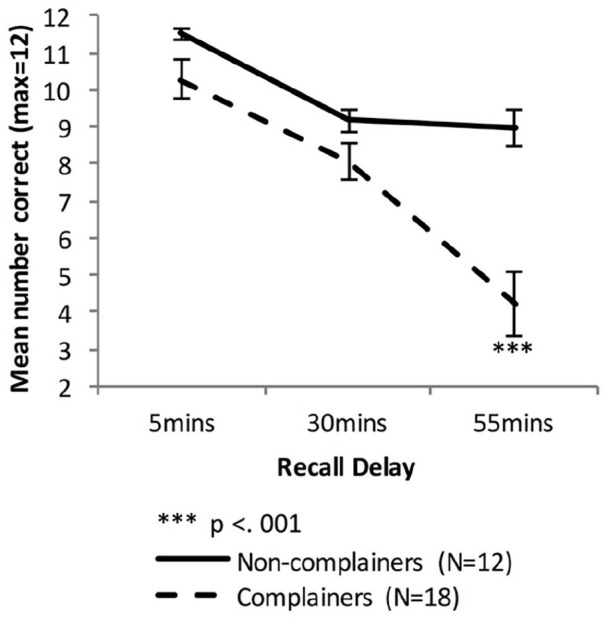
Mean VALMT recall scores as a function time delay and Group, separating the Older group into two groups based on memory complaints (error bars represent one standard error).

Irrespective of initial learning performance (fast or slow), the Older group were now separated into those who reported no subjective memory complaints and those who reported complaints (combining Mild, Moderate, and Severe categories). We then analysed VALMT performance for these two groups. Figure 8 shows the VALMT recall scores for the Non-complainers and Complainers.

A mixed-factors ANOVA with within-subjects factor Delay (5 vs. 30 vs. 55 min) and between-subjects factor Group (Non-Complainers vs. Complainers) was used to analyse cued recall performance across all delay intervals.

There were significant main effects of Delay, (*F*(1.32, 37.0) = 47.99, *p* < .001, η_p_^2^ = .63, BF_10_ = 2.48 × 10^12^, and Group, *F*(1, 28) = 12.48, *p* = .001, η_p_^2^ = .31, BF_10_ = 5,048, and a significant interaction between Delay and Group, *F*(1.32, 37.0) = 11.39, *p* < .001, η_p_^2^ = .29, BF_10_ = 1,314, indicating that compared with the Non-complainers, the Complainers had an overall lower level of recall (Marginal means: *M*_Non-complainers_ = 9.89 pairs, *M*_Complainers_ = 7.52 pairs) and a higher forgetting rate.

To compare cued recall performance across Non-complainers and Complainers at each time-point, three independent sample *t*-tests were used. The difference was significant at 55 min, not significant at 30 min, and at 5 min it was significant before but not after adjusting for multiple comparisons (5 min: *M*_Non-complainers_ = 11.5 pairs, *M*_Complainers_ = 10.3 pairs, *t*(19.5) = 2.15, *p* = .04, Bonferroni adjusted *p* = .12, *d* = 0.68, BF_10_ = 1.12; 30 min: *M*_Non-complainers_ = 9.17 pairs, *M*_Complainers_ = 8.06 pairs, *t*(28) = 1.76, *p* = .09, *d* = 0.68, BF_10_ = 1.09; 55 min: *M*_Non-complainers_ = 9.0 pairs, *M*_Complainers_ = 4.22 pairs, *t*(25.9) = 4.88, Bonferroni adjusted *p* < .001, *d* = 1.64, BF_10_ = 117).

Independent sample *t*-tests found no significant difference in early forgetting rate between the two groups (*M*_Non-complainers_ = 0.20, *M*_Complainers_ = 0.21; *t*(28) = 0.301, *p* = .77, *d* = 0.12, BF_10_ = 0.36), but the Complainers had a significantly higher late-forgetting rate (*M*_Non-complainers_ = 0.01, *M*_Complainers_ = 0.50; *t*(28) = 4.04, Bonferroni adjusted *p* < .001, *d* = 1.55, BF_10_ = 67.79).

#### Relationship between subjective memory complaints and WMS LM scores

**Figure 9.**
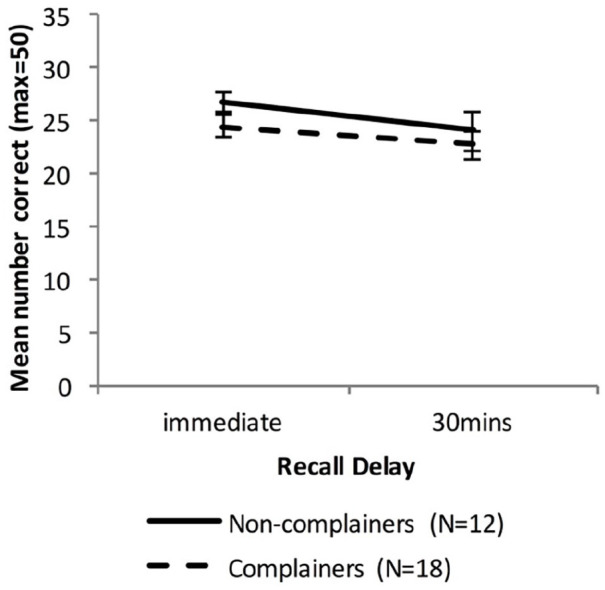
Mean WMS-LM recall scores as a function time delay and Group, separating the Older group into two groups based on memory complaints (error bars represent one standard error).

Figure 9 shows the WMS LM recall scores for the Older group, when split into Non-complainers and Complainers.

A mixed-factors ANOVA with within-subjects factor Delay (immediate vs. 30 min) and between-subjects factor Group (Non-Complainers vs. Complainers) was used to analyse cued recall performance across all delay intervals.

There was a significant main effect of Delay, *F*(1, 28) = 7.50, *p* = .01, η_p_^2^ = .21, BF_10_ = 3.44, but no significant effect of Group, *F*(1, 28) = 1.33, *p* = .26, η_p_^2^ = .04, BF_10_ = 0.55, and no significant interaction between Delay and Group, *F*(1, 28) = 0.30, *p* = .59, η_p_^2^ = .01, BF_10_ = 0.50.

#### Relationship between the Older groups learning performance and subjective memory complaints, response-time, and age

To provide an indication of familiarity with using computers the average time taken to answer each cued-recall query during learning was calculated (“response-time”). The rationale was that participants unfamiliar with computers may also take longer to type their answers, and/or spend more time thinking about what to do. The mean number of trials needed to reach criterion was then correlated with *response-time*, total MCS scores and age, adjusting for multiple comparisons by multiplying the *p*-values by number of comparisons (3).

The correlation of trials to criterion with total MCS scores was large and significant (rho = .554, adjusted *p* = .006), while the correlation with *response-time* was very small and not significant (rho = .041, adjusted *p* = 1.0) and the correlation with age was small and not significant (rho = −.157, adjusted *p* = 1.0).

## Discussion

A pilot study demonstrated that the VALMT word pair learning paradigm developed by McGibbon and Jansari (2013) can reveal differences in forgetting rates between education matched, healthy younger and older individuals within a 55-min delay after learning. As with others (e.g., Davis et al., 2003; Morse, 1993), we found a larger variation in performance in the Older group than in the Younger group, in both learning and recall performance. The greater forgetting seen in the Older group was largely driven by those who required more trials to learn to criterion, or “slow learners,” (Slow_Older), while those who required fewer trials, or “fast_learners,” (Fast_Older) performed very similarly to the Younger group.

Our main study built on these findings, adding additional experimental controls, adding measures for subjective memory complaints and sleep quality, adding a standardised anterograde memory test for comparison, and matching for IQ. In agreement with our prediction and the results from the pilot, the overall analysis showed that while the Younger group showed a very shallow forgetting over the period of 55 min, the combined Older group showed a steeper forgetting function, with the difference in performance reaching statistical significance by 55 min. When the Older group was separated based on initial learning rate (number of trials), different patterns of forgetting were again revealed. As in the pilot study, the fast learning Fast_Older group performed similarly to the Younger group at all delays, while the slow learning Slow_Older group demonstrated lower recall at all time points, and a faster rate of forgetting between 30 and 55 min. Importantly, performance on a standardised assessment (WMS-LM) suggested that none of these healthy participants had a memory impairment that would be diagnosed using this existing clinical measure.

As the VALMT is a new paradigm, it is important to understand how sensitive it is in comparison to existing standard clinical measures. To evaluate this, a comparison with the WMS-LM test was performed. As the WMS-LM measures recall immediately after learning and after a 30-min delay, it was not possible to compare performance with the VALMT at the longer 55-min delay. Over the initial 30 min, the WMS-LM results for the combined Older group matched the VALMT results: no statistical difference was found between Younger and Older groups in either recall performance or forgetting rate. However, once the Older group was split into Fast and Slow learners a clear difference appeared. At 30 min WMS-LM was unable to identify any difference between the Younger, Fast_Older and Slow_Older groups, while the VALMT identified lower recall performance for the Slow_Older group, indicating that the VALMT is able to reveal rapid forgetting in a manner that a standard clinical measure is unable to. The VALMT 55 min results show a further large drop in Slow_Older performance and a significantly accelerated late forgetting rate (30–55 min).

Group differences in self-reported sleep quality were small, and not statistically significant. However, they did follow the same ordinal pattern as recall performance, with the Slow_Older group showing the worst sleep quality, followed by Fast_Older and then Younger groups. One possible explanation for the lack of a significant difference may be the slightly younger age of our Older group (60–69 years) in comparison to those in other studies such as Mary et al. (2013; 65–75 years).

By contrast, the group differences in self-reported memory complaints were large, and statistically significant. The combined Older group reported more memory complaints than the Younger group, with a large effect size. When the Older group was split into Fast and Slow learners, the analysis showed that the bulk of the difference in memory complaints was driven by the Slow_Older group. When the combined Older group was split into Complainers and Non-complainers, there was a clear difference in VALMT performance. The Complainers scored significantly lower at 55 min, and had a higher late forgetting rate (30–55 min). This provides strong evidence of a link between subjective memory complaints and objective cued-recall performance as measured by the VALMT. The WMS-LM, by contrast, was unable to differentiate between these groups, with no difference in recall scores or forgetting rate, although it should be noted the longest delay for this test was 30 min, not 55 min. This suggests that the VALMT may be better able to identify subtle objective memory differences that are linked to subjective complaints, although a comprehensive comparison against WMS-LM will require future testing at 55-min delay.

Before reviewing key issues raised by these results we would like to clarify the terminology. First, initial definitions of ALF included normal learning as a requirement. This eliminates learning performance as a cause of later differences in forgetting rates, and therefore provides the best theoretical justification for the existence of ALF. However, we agree with Baddeley and colleagues (Baddeley et al., 2014, 2021; Laverick et al., 2021) that there is no reason to believe that accelerated forgetting at later delays cannot be present in those whose capacity for learning is impaired, and that insistence on normal learning performance may have led to under-reporting of ALF. Laverick et al. suggest that use of another term may be appropriate where learning is not normal, for example, “Speeded Long-term Forgetting” (SLF). For this discussion, we will use the standard term, ALF, but in the more general sense that includes cases where learning performance is impaired. Second, we will use “accelerated forgetting” to mean a rate of forgetting that is increasing in comparison to that of a control or comparison group. For example, if the forgetting rate for the control group slows over time while a patients group continues in a linear fashion, we would class this as accelerated forgetting as the difference in group forgetting rates increases with time. We believe most ALF literature uses the term in this way.

### Why did we identify differences in performance at shorter timeframes than previous studies?

How is it that healthy older individuals who successfully learnt material to criterion, and were not impaired on a standard clinical measure of memory, show significant impairment on the VALMT within 55 min?

Although our participants report no diagnosed psychological or medical conditions that affect memory, perhaps some have an undiagnosed medical condition of this type, in particular, AD or another cause of dementia. As we were unable to administer a cognitive screening tool, such as the mini-mental state exam (MMSE), this remains a possibility. However, episodic memory is generally the first mental capacity to be impacted by such conditions, and therefore the fact that all our participants perform normally on the WMS-LM, a standard test of anterograde memory, argues against this. For future work the use of a standardised cognitive screening tool would be beneficial.

In the Manes et al. (2008) study, the Older “Complaint” group who showed accelerated forgetting at 6 weeks was unimpaired at 30 min on their story recall task This mirrors the pattern seen in our WMS-LM results, where we found no difference between the groups at immediate recall or 30 min. It may be that story-recall performance would differ between groups if tested at the longer 55-min delay. However, a more likely explanation seems to be to the type of task.

Due to the “scaffolding” that story grammars provide, recalling such material is generally an easier task than remembering word pairs that are unrelated. This may well have contributed to the normal performance of the Manes et al. (2008) Complaint group at 30 min. Indeed, McGibbon and Jansari (2013) have demonstrated that their patient with subclinical epilepsy could pass the standard tests of memory as well as a story recall task at 30 min (Jansari et al., 2010), but still show a significant impairment on their word pair learning task by 55 min. Further evidence for this argument comes from the comparison of VALMT and WMS-LM scores, with the story recall based WMS-LM showing no group differences, while the VALMT identified significant differences in both recall levels at all time points (5, 30 and 55 min) and late forgetting rate (30–55 min).

In common with Manes et al. (2008), Baddeley et al. (2014) did not find a significant difference until 6 weeks when comparing Younger and Older participants. Their task used cued-recall of events, each detailed in 3 or 4 sentences (“constrained prose”; “Crimes Test”). At each delay, they tested recall for a separate subset of items from each event, to avoid repeated recall. Although the test involved answering questions about each event rather than freely recalling it, the argument that “scaffolding” with integrated material makes the task easier would apply. Although they did test at intermediate intervals varying between 24 hr and 24 days, they failed to find any significant differences at these time points. However, only eight participants were tested at each intermediate delay, which they acknowledge may have contributed to the failure to find significant forgetting at these shorter delays. Furthermore, in later work Baddeley et al. (2019) found evidence that although their Crimes Test procedure evaluates recall of different elements of each event at each delay (to avoid repeated recall), this partial recall was priming the other elements of each event, resulting in reduced forgetting of these non-tested elements. In follow-up work using a modified procedure with recall of separate unrelated events at each delay (Baddeley et al., 2021), they found this priming was avoided and forgetting was more rapid. It is possible that using this modified procedure might lead to detection of ALF in older populations at shorter delays than 6 weeks.

In addition to facilitating delayed recall, the integrated nature of materials such as short stories may also hide learning deficits in Older participants. Naveh-Benjamin et al. (2003) show that ageing impacts formation of associations between arbitrary items (their “Associative Deficit Hypothesis,” ADH), and that this associative deficit is reduced when the components of the episode are already connected in memory, such as the case with coherent stories or semantically related words. In contrast, the use of semantically unrelated word pairs, as in VALMT, would be expected to highlight any such deficit.

Baddeley et al. (2014) rightly point out that story recall is a more naturalistic paradigm which is useful for looking at long-term memory issues. However, from a clinical perspective it is desirable to obtain objective evidence within a limited amount of time, preferably within a single clinical session, and given the arguments above this can be difficult with story recall. By contrast, importantly, our results suggest that it may be possible to obtain such evidence within a single visit using the VALMT.

In the Mary et al. (2013) study, the Older group was also unimpaired at the 30-min interval on their task, which like ours was also a word pair learning task. However, in their paradigm, to help participants develop associations between the unrelated words, diagrams depicting the words were presented and participants were actively encouraged to use mental imagery to associate the words together. Furthermore, the Older participants were exposed to the stimuli for twice as long as the Younger participants. As both mental imagery (Hussey et al., 2012; Sheldon et al., 2017) and increased exposure times to materials (e.g., Ganor-Stern et al., 1998) are known to improve retention, these factors will have strengthened memory for the Older group, which may have masked any difference between groups at the 30-min interval.

A further possible explanation for the early differences we identified in delayed recall performance may be interference; specifically, differences in the amount of interference groups are exposed to, or differences in groups’ vulnerability to interference, or a combination of these factors. The VALMT procedure can introduce interference in two main ways.

First, interference may be introduced within a single learning period (learning a set of 12 pairs). Once a pair has been learnt to criterion it stops being represented, while testing and presentation of the remaining pairs continues. This may create interference towards the previously learnt pairs. In addition, the errors made during unsuccessful recalls of a given pair may create interference for that pair. Indeed, there is some evidence that using procedures that eliminate errors during learning (“errorless learning”) can be beneficial for healthy elderly (Baddeley & Wilson, 1994; Wilson et al., 1994) and effective as a memory rehabilitation technique for AD patients (Clare et al., 2002). Both of these potential sources of interference within a learning period will be worse for slow learners who require more trials overall. Note that a “trial” in VALMT refers to an attempt to recall a single pair, whereas a trial in most other paradigms refers to an attempt to recall an entire list of words.

Second, the overall VALMT procedure used in this study is relatively complex and involves interleaving of multiple learning and testing phases. The additional learning and testing activities that occur between learning and delayed test of a given pair may create interference. Any such interference will be greatest for material recalled at 55 min and least for material recalled at 5 min, and again will be worse for slow learners who require more trials over all.

To empirically investigate the effects of interference the delayed recall results for pairs recalled at 55-min pairs were broken down by learning period. One-third of these pairs are learnt during Learning Period 1 (L3a), one-third during Learning Period 2 (L3b), and one-third during Learning Period 3 (L3c). Potential interference should be greatest for those learnt during Learning Period 1, intermediate for those learnt during Period 2, and smallest for those learnt during Period 3 (refer to Figure 1 for detail). By comparing the recall results for these subsets, we investigated the impact of interference induced by the procedure. If interference was impacting the results, we would expect to have found L3a getting the lowest recall score (as it encounters the greatest potential interference), L3b the second highest, and L3c the highest score. In fact, we found no statistically significant main effect of learning period, although the BF indicates that the data provide only anecdotal evidence for a lack of effect. However, we found strong evidence for a lack of an interaction between learning period and group (Younger, Fast Older, Slow Older), indicating that any interference does not impact our groups differently. Taken together, this suggests inconclusive evidence that interference is not a cause of forgetting between delays, and strong evidence that it is not the cause of the observed group differences in forgetting.

While these data argue against interference as a primary driver of our results, further work will be required to fully validate this. For example, future work could include a condition in which participants learn a single set of 12 pairs in one learning period and recall these after 55 min, with the rest of the procedure dropped. This would eliminate interference due to the interleaving of learning and testing, providing a valuable comparison. In addition, planned enhancements to the VALMT software will record the point at which each word pair reaches criterion during the learning process, facilitating granular analysis of possible interference within a learning period.

If future work indicates that interference is, in fact, a major driver of results, then providing a confound-free measure of forgetting, especially in the presence of a learning deficit and therefore differential interference, will require further development of the VALMT procedure.

A comparison with existing clinical tests highlights a trade-off in design: the VALMT equates learning and avoids differential over-learning and retrieval practice but may introduce differential interference, while many clinical tests hold the number of presentations fixed (e.g., CVLT, Delis et al., 1987; WMS-LM), which limits differential interference, but learning is not equated since material is not learnt to criterion and differential overlearning and retrieval practice can occur. The potential role of interference due to learning errors also highlights the need to record and analyse learning error rates. Many ALF studies use free-recall of a list of words as a measure of verbal memory (e.g., Butler et al., 2007; Gascoigne et al., 2012; Mameniskiene et al., 2006; Weston et al., 2018). In such studies, the number of times the entire list is presented is recorded and analysed as a measure of learning, but the number of individual errors made (recalling a word which was not on the list) is not. Similarly, where word pairs are used as stimuli (e.g., Atherton et al., 2014; Mary et al., 2013) the number of times the entire list is presented is reported and analysed, but not the number of incorrect responses given. It may be that these incorrect responses made during learning are influencing the results of such studies, and by not recording and analysing these, a subtle learning deficit has been missed. If that is the case, then later forgetting identified as ALF in the strong sense of the term may not in fact reflect a pure retention problem.

Slower learners will also take more time in total to complete the full VALMT procedure. It is thus possible that they will experience greater fatigue, and that this may influence their results. The interleaving of learning and testing means that any increased fatigue should impact performance at all test delays. Any impact might therefore be expected to reduce scores at all delays for slow learners, rather than influencing the rate of forgetting between delays. Thus, it is hard to see how differences in fatigue could be the primary cause of the large difference in forgetting rates seen between 30 and 55 min. More conclusive evidence could be provided by the previously suggested future replication, including a condition in which participants learn a single set of 12 pairs in one learning period and recall these after 55 min with the rest of the procedure dropped. This would greatly reduce any possibility of differential fatigue.

Finally, in the case of the VALMT, as individual pairs are only presented until they have been learnt to criterion, the pairs which are learnt faster will also experience a longer duration delay before delayed testing, compared with those which are learnt more slowly, which adds a confound, and again this would be greater for slower learners. However, in practice the maximum extra delay introduced is of the order of tens of seconds. While this might influence recall at the 5-min delay, any impact will be negligible at the 30- and 55-min delays.

Overall, the differences between the highlighted studies demonstrate the sensitivity of different types of paradigms for revealing subtle issues, and further demonstrate the impact of the different methodologies used (Elliott et al., 2014).

### Why do some participants show poorer learning performance, and what is the significance of this?

Why do some Older participants take significantly more trials to complete learning to criterion? What could be causing their slower learning?

First, it could be caused by the early preclinical stages of AD, or some other age-related cognitive decline. While this study did not include measurement of any AD biomarkers, it is known that memory complaints are a predictor of future development of MCI and AD (Mitchell et al., 2014; Weston et al., 2018). If slow learning was caused by an early stage AD process, we would expect slow learners to report a higher level of memory complaints. This is indeed what was observed, with the correlation being large and significant.

Second, it may be that our Older participants were less familiar with using computers, which may make the computer-based learning process harder for them in some way and thus lead to more errors. However, all participants completed a demonstration before the main study started; they were exposed to the software and could ask questions of the researcher. They were asked if they understood the task before proceeding. This would argue against familiarity with computers being the main cause of errors. Participants unfamiliar with computers may also take longer to type their answers, and/or spend more time thinking about what to do. However, the mean seconds per response during learning did not correlate with the number of trials needed to learn to criterion, which also suggests familiarity with computers was not the primary cause.

Third, some of our Older participants may have paid less attention, or may have invested less effort. While there was no direct attention measure built into the task or procedure, the fact that testing was done face to face with a researcher present, and the researcher did not observe any noticeable lack of attention, suggests that participants are likely to have paid attention to what they were doing.

Fourth, some of our Older participants may have found it harder to understand or follow instructions. However, the fact that there were no differences in IQ or education between our fast and slow-learning groups suggests that this is unlikely.

Finally, slower learning could be directly age related, in which case Older participants should make more errors. However, there was no correlation between age and learning performance in our Older group.

Overall, the evidence suggests that the slower learning some Older participants displayed is caused by a cognitive deficit, in which case it is possible that VALMT learning performance provides an indicator of preclinical AD or another form of dementia. Further investigation of this possibility should include use of a cognitive screening tool such as MMSE to eliminate any possibility of undiagnosed medical conditions which could affect memory, combined with longitudinal follow-up to validate the success of VALMT in predicting progression to MCI or AD.

The larger variation seen in learning performance in our Older group also reminds us that while matching individuals on the main demographic variables is the standard procedure in research where overall group performance is under investigation, in neuropsychology we should also consider individual differences as they can impact performance significantly.

### Is there evidence for different forgetting curves, and does forgetting start early or later?

Whereas early theories of memory formation referred to one process of consolidation to stabilise engrams, more recent formulations have suggested a more complex process. For example, Dudai (2004) has differentiated between an early process known as “synaptic consolidation” which occurs in the first few minutes to possibly hours in the hippocampal complex, and “systems consolidation” which occurs over long time frames and although initially dependent on the hippocampus, becomes independent of that region over time. This has led some researchers to compare rates of forgetting at two different time points. For example, Hoefeijzers et al. (2013) compared forgetting in the first 30 min with that between 30 min and 1 week later, and found that their TEA patients were equivalent to their controls in terms of the “early forgetting” but significantly different for the “late forgetting.” They used this difference to argue that the ALF that the patients experienced was caused by a consolidation problem and, in particular, late consolidation. They do concede, however, that “forgetting across [the] early time interval is rather limited in both groups. This relative lack of early forgetting may be the reason we did not find a correlation between early and late forgetting rates” (Hoefeijzers et al., 2013, p. 1554).

Many other studies in the ALF literature which report late-onset forgetting were performed with TLE patients (e.g., Mameniskiene et al., 2006). However, in a study with TLE patients which addressed several methodological issues identified in previous work, Cassel et al. (2016) found that forgetting was detectable by 10 min in their visuo-spatial task, while for short stories they found forgetting developed in a more progressive manner, starting early but only reaching statistical significance after 1 week. Cassel et al. (2016) interpreted their findings as evidence for forgetting starting during the early consolidation stage, and suggest that differences in forgetting patterns reflect a continuum of severity and/or sensitivity rather than a difference between early and late-onset forgetting.

While the VALMT uses word pair learning rather than story recall, the methodology is closer in design to that of Cassel et al. (2016) in that it requires learning to criterion across all individual word pairs. A further similarity is that the current study used different word pairs for testing at different time points. Both these factors help minimise the potential problem of retrieval practice.

Our Slow_Older group scored lower that our Younger and Fast_Older groups at all time points, even at 5 and 30 min. They also showed a similar early forgetting rate (5–30 min) to the other two groups, but then an accelerated late forgetting rate (30–55 min). Taken together, the lower recall at early delays combined with an accelerated forgetting seem to more strongly support an early-onset and progressively increasing forgetting pattern rather than a late-onset forgetting in our sample. However, even if ALF in TLE and in our Slow_Older group reflects early-onset forgetting, it remains possible that other participants or groups may show more pronounced forgetting at longer intervals of days or even weeks.

In a large review of forgetting rate studies, Radvansky et al. (2022) found that forgetting does not follow a single forgetting function, as usually assumed. They found evidence of acceleration and deceleration of forgetting at different timescales. They propose a “Memory Phases Framework” with four intervals based on cognitive and neurobiological evidence: up to 60 s (working memory), 60 s–12 hr (early long-term memory, eLTM), 12 hr–7 days (transitional long-term memory, tLTM), and beyond 7 days (long-lasting memory, LLM). They found that forgetting rate increases during the WM phase, slows during eLTM, remains relatively stable during tLTM and increases again in LLM.

The current study falls within the eLTM phase, where forgetting rate should decrease as hippocampal consolidation occurs. Our results show this standard pattern for our Younger and Fast_Older groups but not for our Slow_Older group, suggesting hippocampal consolidation is impaired in our Slow–Older participants. Whether there remain others within our sample who show normal forgetting to 55 min as measured by the VALMT but would then go on to display greater forgetting during the tLTM or LLM phases, perhaps due to problems in neocortical consolidation or retention, while theoretically plausible, remains an open question. The VALMT as currently designed specifically targets early identification of memory deficits, within a single clinical visit. It could be adapted to monitor memory over delays of days or weeks, or alternatively the Crimes and Four Doors tests developed by Baddeley and colleagues (Baddeley et al., 2014, 2021; Laverick et al., 2021) may be more appropriate for such extended delays. Future comparisons would help clarify whether a single test can address all timeframes adequately, or whether multiple tests are required.

### Is the VALMT suited to early identification of those at risk of developing MCI or AD?

Those who subjectively report suffering from memory problems can often perform normally on standardised clinical memory assessments which measure performance at delays of up to 30 min. Many explanations have been proposed for this inconsistency. One possible explanation is that subjective complaints reflect forgetting that occurs over a timeframe beyond that tested with standard clinical measures, which aligns with the late-onset forgetting conception of ALF (Manes et al., 2008; McGibbon & Jansari, 2013). Another possible explanation is that existing clinical measures are not sensitive enough to spot subtle early impairments which are the underlying cause of the complaints, which aligns better with the early-onset progressive forgetting conception of ALF (Cassel et al., 2016).

We found no relationship between complaints and recall performance for the WMS-LM tests, but a strong correlation with VALMT scores. Combined with the recall impairments seen with the VALMT, this provides evidence for an early-onset of forgetting. This also suggests that the VALMT (testing at delays up to 55 min) is a more sensitive test than the WMS-LM (testing at the standard 30 min) at detecting this and can, within the window of a single clinical visit, detect subtle impairments that are related to memory complaints, although a more conclusive comparison between the two tests will require future work testing with the WMS-LM at a 55-min delay. This is important in relation to MCI and AD, as there is evidence that those who perform normally on standard tests but complain of memory problems are at greater risk of going on to develop MCI and AD. For example, a meta-analysis by Mitchell et al. (2014) found that older people with subjective memory complaints who perform normally on objective tests are twice as likely to develop dementia as individuals without complaints. Similarly, Weston et al. (2018) found that subjective memory complaints increased in presymptomatic autosomal dominant (familial) AD as participants approached expected onset of symptoms.

There are other reasons to predict that the VALMT may be suitable for early detection of those at risk of MCI/AD. One of the first cognitive functions impacted by AD is episodic memory (Fox et al., 1998), and impairment of this sort is used to diagnose amnestic MCI (aMCI) which carries an increased risk of progression to AD (Silva et al., 2012). To reliably detect such impairment at an early stage it would be advisable to use a test that relies heavily on memory and as little as possible on use of cognitive strategies. Cued-recall provides some support for recall by means of the cue-word, reducing the need for recall strategies. In addition, the use of unrelated word pairs makes it harder to use strategies based on word categories, and avoids the scaffolding provided in story recall paradigms. Together, these elements of the VALMT reduce confounding that could be introduced by cognitive strategies and should make this test well suited to detection of early stage episodic memory impairment.

Furthermore, associative learning of the type used in the VALMT is vulnerable to the impact of early stage AD (Sapkota et al., 2017). Associative learning is also known to rely on entorhinal cortex and hippocampal brain regions which are vulnerable to change in early AD (Coupé et al., 2019; de Rover et al., 2011). So, it is to be expected that early stage AD would impact VALMT scores.

Finally, if the disease process has led to subtle learning deficits, the more challenging VALMT learning process which requires learning each pair to criterion may provide a more sensitive test than existing clinical tests such as the CVLT and WMS-LM which use a fixed number of presentations and do not require learning to criterion.

### Limitations and future direction

There are some limitations to our study that future studies could address. First, while our sample sizes are equivalent to those of some similar studies (e.g., Manes et al., 2008), they are nonetheless modest and so future studies on larger samples are needed to replicate our findings.

Second, we have compared the VALMT with the story-recall based WMS-LM test. It would be useful to extend the comparison to other forms of verbal test such as word-list recall (e.g., California Verbal Learning Test) and other paired-associate tests (e.g., WMS Verbal Paired Associate). It would also be useful to investigate use of these standardised tests at 55 min, to match the longer VALMT delay.

Third, future studies could investigate adapting the VALMT to add a measure of forgetting which avoids any possible confound from differential interference, whether due to some participants requiring more trials and making more errors during learning, or the interleaving of learning and testing.

Fourth, it would be beneficial to validate these results in additional languages. The VALMT has already been translated into a number of other languages and replications are already underway.

Finally, while difficult to realise, it would be useful to longitudinally follow-up the Older group, in particular the slow learners, to validate whether the VALMT can predict progression to aMCI or AD.

## Conclusion

In sum, the current study has used a variant of the VALMT word pair learning paradigm to reveal differences in delayed recall performance between younger and healthy Older participants at an earlier time point than documented previously. Importantly, we have shown that those Older participants who learn more slowly also forget more rapidly within the window of a standard clinical visit, a difference which a standardised clinical measure was unable to detect. This objective impairment of the slow learners is associated with increased subjective memory complaints.

The study highlights both the importance of the specific details of the methodology and the need for taking into consideration the variance between individuals that only begins to express itself as we age and which may be the underlying basis for why some people forget rapidly and others do not.

Further studies addressing these fine-grained issues are needed to elucidate the mechanisms of accelerated forgetting with ageing, as well as to support the pursuit of a clinically reliable test for objectively capturing this forgetting. The effectiveness of such sensitive tests in predicting future progression to MCI and AD is also worthy of future investigation.

## Supplemental Material

sj-docx-1-qjp-10.1177_17470218221113412 – Supplemental material for Accelerated forgetting in healthy older samples: Implications for methodology, future ageing studies, and early identification of risk of dementia Click here for additional data file. Supplemental material, sj-docx-1-qjp-10.1177_17470218221113412 for Accelerated forgetting in healthy older samples: Implications for methodology, future ageing studies, and early identification of risk of dementia by Terence McGibbon, Ashok Jansari, Jessica Demirjian, Ana Nemes and Adrian Opre in Quarterly Journal of Experimental Psychology
