# Supplementary material for: Accelerated forgetting in healthy older samples: Implications for methodology, future ageing studies, and early identification of risk of dementia
Source: Q J Exp Psychol (Hove). 2022 Aug 1;76(6):1347–67. doi: 10.1177/17470218221113412 (PMC10196925; doi:10.1177/17470218221113412)
Supplement: sj-docx-1-qjp-10.1177_17470218221113412 – Supplemental material for Accelerated forgetting in healthy older samples: Implications for methodology, future ageing studies, and early identification of risk of dementia [file sj-docx-1-qjp-10.1177_17470218221113412.docx]

Supplementary Material for:

**Accelerated forgetting in healthy older samples: implications for methodology, future ageing studies and early identification of risk of dementia.**

Terence McGibbon, Ashok Jansari, Jessica Demirjian, Ana Nemes & Adrian Opre

**Pilot study**

***Participants***

Two groups of participants were assessed: 43 Younger participants aged 20-30 (24F, 19M: Mean Age: 22.84, SD: 2.46) were compared to 26 Older participants aged 65-80 (16F, 10M: Mean Age: 70.62, SD: 4.71). Due to a technical error learning performance data was unavailable for 7 Older participants. Analysis with sub-groups based on learning performance analysis was therefore conducted with 19 Older participants.

***Procedure and materials***

The VALMT procedure and materials were the same as those detailed in the main study, except that materials and instructions were translated into Romanian. Unlike the main study, due to limits on time and resources no standard neuropsychological tests were performed, and no data was gathered on subjective memory complaints or sleep patterns.

***Results***

Figure A1 shows the delayed recall performance of the Younger group and the combined Older group. Figure A2 shows the performance with Older group split into fast and slow learners based on learning trials required to reach criterion (Fast Older, Slow Older), implemented due to an apparent bimodal distribution in this variable.

*Figure A1. Pilot study: Mean VALMT recall scores as a function of time delay and group (error bars represent one standard error)*

*Figure A2. Pilot study: Mean VALMT recall scores as a function time delay and group, separating the Older group into two groups based on initial learning (error bars represent one standard error)*

*Combined Older group compared to Younger group*

A mixed factors ANOVA with within-subjects factor Delay (5min vs 30min vs 55min) and between-subjects factor Group (Younger vs Older) was used to analyse cued recall performance across all delay intervals. Significant main effects of Delay (*F*(2, 121) = 69.08, *p* < .001, *η_p_^2^* = 0.51, BF_10_ = 3.34*10^14^) and Group (*F*(1, 67) = 40.71, *p* < .001, *η_p_^2^* = 0.34, BF_10_ = 2.84*10^8^) were found, along with a significant interaction (*F*(2, 121) = 11.89, *p* < .001, *η_p_^2^* = 0.15, BF_10_ = 3100).

Independent samples t-tests were used to compare recall performance between groups at each delay interval. The Older group scored significantly lower than the Younger group at all 3 delay intervals; 5mins (*M*_Older_ = 10.54 pairs, *M*_Younger_ = 11.81 pairs; *t*(31.2) = 4.71, *p* < .001, *d* = 1.38, BF_10_ = 25511), 30mins (*M*_Older_ = 8.77 pairs, *M*_Younger_ = 10.88 pairs; *t*(35.8) = 3.91, *p* < .001, *d* = 1.09, BF_10_ = 519) and 55mins (*M*_Older_ = 6.54 pairs, *M*_Younger_ = 10.14 pairs; *t*(67) = 6.07, *p* < .001, *d* = 1.51, BF_10_ = 1.20*10^5^).

Forgetting rates were calculated as amount of information lost between two consecutive time points relative to the amount that had been recalled at the earlier of the two time points. Therefore, the ‘early’ forgetting rate (that between the 5 and 30 minute time points) was calculated as [5min score – 30min score]/5min score, and the ‘late’ forgetting rate (that between the 30 and 55 minute time points) was calculated as [30min score – 55min score] / 30min score. Independent samples t-tests found the Older group had a significantly greater early-forgetting rate (*M*_Older_ = .17, *M*_Younger_ = .08; *t*(34.3) = 2.15, *p* = .039, *d* = 0.61, BF_10_ = 3.11) and late forgetting rate (*M*_Older_ = .25, *M*_Younger_ = .06; *t*(67) = 3.53, *p* = .001, *d* = 0.88, BF_10_ = 38.38).

The analysis above uses all Older participants (*N* = 26), including those for whom learning data was not available. For comparison, the analysis was repeated using only those Older participants for whom learning data was available (*N* = 19). All tests of statistical significance produced the same result using this smaller group.

*Fast and slow learning Older groups*

A mixed factors ANOVA with within-subjects factor Delay (5min vs 30min vs 55min) and between-subjects factor Group (Younger vs Fast_Older vs Slow_Older) identified significant main effects of Delay (*F*(2,118) = 56.94, *p* < .001, *η_p_^2^* = 0.49, BF_10_ = 2.31*10^14^) and Group (*F*(2,59) = 48.08, *p* < .001, *η_p_^2^* = 0.60, BF_10_ = 7.17*10^9^), and a significant interaction (*F*(4,118) = 6.93, *p* < .001, *η_p_^2^* = 0.19, BF_10_ = 1337). Bonferroni post hoc tests found no significant difference between the Younger and Fast_Older group (*p* = .195, BF_10_ = 1.83), but significant differences between the Younger and Slow_Older (*p* < .001, BF_10_ = 1.61*10^15^) and importantly, between the Fast_Older and Slow_Older (*p* < .001, BF_10_ = 2383).

Recall scores at each delay were compared using one-way ANOVAs with Bonferroni post hoc tests of significant results. There was a significant difference between the means at all 3 delays (5mins: *F*(2,59) = 33.63, *p* < .001, *η_p_^2^* = 0.53, BF_10_ = 4.45*10^7^; 30mins: *F*(2,59) = 27.25, *p* < .001, *η_p_^2^* = 0.48, BF_10_ = 1.96*10^6^; 55mins: *F*(2,59) = 26.46, *p* < .001, *η_p_^2^* = 0.47, BF_10_ = 1.85*10^6^). At all delays the Slow_Older group performed statistically below the Younger (5min: *p* < .001, BF_10_ = 3.76*10^8^; 30min, *p* < .001, BF_10_ = 9.80*10^5^; 55min: *p* < .001, BF_10_ = 1.95*10^6^) and Fast_Older groups (5min: *p* < .001, BF_10_ = 12.39; 30min, *p* < .001, BF_10_ = 151; 55min: *p* = .002, BF_10_ = 32.48). There was no significant difference between the Younger and Fast_older groups’ performance at any delay (5min: *p* = .281, BF_10_ = 1.43^6^; 30min, *p* = 1.00, BF_10_ = 0.35; 55min: *p* = 0.063, BF_10_ = 2.56).

Forgetting rates were compared across groups using one-way ANOVAs. The difference between the means approached significance for early forgetting (Welch’s *F*(2,13.6) = 3.68 *p* = .053, *η_p_^2^* = 0.24, BF_10_ = 65.02) and was significant for late forgetting (*F*(2,59) = 5.71, *p* = 0.005, *η_p_^2^* = 0.16, BF_10_ = 10.83). Bonferroni post hoc tests found the Slow_Older group had faster early and late forgetting rates than the Younger group (early: *p* = .001, BF_10_ = 93.74; late: *p* = .013, BF_10_ = 7.88), and higher early, but not late, forgetting rates than the Fast_Older group (early: *p* = .002, BF_10_ = 3.60; late: *p* = 1.00, BF_10_ = 0.46). There was no significant difference between the Younger and Fast_Older groups’ early or late forgetting rates (early: *p* = 1.00, BF_10_ = 0.47; late: *p* = .108, BF_10_ = 1.81).
